# Mebendazole-induced M1 polarisation of THP-1 macrophages may involve DYRK1B inhibition

**DOI:** 10.1186/s13104-019-4273-5

**Published:** 2019-04-22

**Authors:** Kristin Blom, Jenny Rubin, Malin Berglund, Malin Jarvius, Lena Lenhammar, Vendela Parrow, Claes Andersson, Angelica Loskog, Mårten Fryknäs, Peter Nygren, Rolf Larsson

**Affiliations:** 1 Department of Medical Sciences, Division of Cancer Pharmacology and Computational Medicine, Uppsala University, 75185 Uppsala, Sweden; 2 Department of Immunology, Genetics and Pathology, Uppsala University, 75185 Uppsala, Sweden

**Keywords:** Monocytes and macrophages, Mebendazole, M1 polarisation, DYRK1B

## Abstract

**Objective:**

We recently showed that the anti-helminthic compound mebendazole (MBZ) has immunomodulating activity by inducing a M2 to M1 phenotype switch in monocyte/macrophage models. In the present study we investigated the potential role of protein kinases in mediating this effect.

**Results:**

MBZ potently binds and inhibits Dual specificity tyrosine-phosphorylation-regulated kinase 1B (DYRK1B) with a Kd and an IC_50_ of 7 and 360 nM, respectively. The specific DYRK1B inhibitor AZ191 did not mimic the cytokine release profile of MBZ in untreated THP-1 monocytes. However, in THP-1 cells differentiated into macrophages, AZ191 strongly induced a pro-inflammatory cytokine release pattern similar to MBZ and LPS/IFNγ. Furthermore, like MBZ, AZ191 increased the expression of the M1 marker CD80 and decreased the M2 marker CD163 in THP-1 macrophages. In this model, AZ191 also increased phospho-ERK activity although to a lesser extent compared to MBZ. Taken together, the results demonstrate that DYRK1B inhibition could, at least partly, recapitulate immune responses induced by MBZ. Hence, DYRK1B inhibition induced by MBZ may be part of the mechanism of action to switch M2 to M1 macrophages.

**Electronic supplementary material:**

The online version of this article (10.1186/s13104-019-4273-5) contains supplementary material, which is available to authorized users.

## Introduction

Mebendazole (MBZ), a drug commonly prescribed for various forms of helminthic diseases has demonstrated anticancer activity in several in vitro and in vivo model systems [1–11]. In the clinical setting MBZ has also induced tumour responses in therapy-resistant patients with adrenocortical and colorectal cancer [12, 13].

The primary anticancer mechanism of MBZ has been attributed to its ability to target and inhibit tubulin polymerization in tumour cells [3, 4]. However, other mechanisms, such as angiogenesis inhibition [6, 9], apoptosis induction [2, 8] and inhibition of the Hedgehog signalling pathway [14] have been proposed. Moreover, MBZ has also been shown to potently bind to several protein kinases involved in oncogenic signaling [7].

Recently, we showed that MBZ induce a pro-inflammatory tumour-suppressive M1 phenotype in THP-1 monocytes and macrophages which could potentially explain tumour cell killing [15]. We also recently demonstrated that MBZ potentiate the anti-cancer activity of CD3/IL2 activated peripheral blood mononuclear cells (PBMCs) and that this effect was attenuated by removal of CD14+ myeloid cells [16]. Hence, MBZ is seemingly an interesting small molecule drug to tilt the M2 rich tumour microenvironment in favour of M1 macrophage differentiation, which in turn may induce anti-tumour immunity.

In the present study, we investigated the possible role of protein kinases for the immune modulating properties of MBZ in THP-1 monocytes and macrophages. The results show that DYRK1B inhibition could, at least partly, mimic the immune responses induced by MBZ and could be part of the mechanism of action for its macrophage M1 polarisation.

## Main text

### Methods

#### Materials

MBZ, AZ191, lipopolysaccharide (LPS), interferon gamma (IFNγ), interleukin-13 (IL13), phorbol-12-myristate-13-acetate (PMA) and interleukin-4 (IL4) were purchased from Sigma Aldrich (Sigma, St. Louis, MO, USA). The compounds were stored as 10 mM stock solutions in dimethylsulfoxide (DMSO, Honeywell, Morris Plains, NJ, USA) or sterile water and further diluted with culture medium (see below).

#### Cell culture

Cell culture was performed as previously described [15]. Monocytoid THP-1 cells were purchased from American Type Culture Collection (ATCC; Manassas, VA, USA) and were cultured in RPMI-1640 medium supplemented with 10% heat-inactivated fetal bovine serum, l-glutamine (2 mM), penicillin/streptomycin (100 U/100 µg/mL) and 0.05 mM 2-mercaptoethanol (Sigma). The cell line was cultured at 37 °C in a humidified atmosphere with 5% CO_2_. For the differentiation and polarisation of THP-1 cells to macrophages, PMA (97.2 nM), IFNγ (20 ng/mL), LPS (100 ng/mL), IL4 (20 ng/mL) and 20 ng/mL IL13 (final concentrations) were used according to an established protocol with minor modifications [17]. PMA was added to THP-1 cells seeded into wells of a 12-well plate (Corning). After 30 h incubation, LPS/IFNγ (M1), IL4/IL13 (M2), DMSO (control), AZ191 and MBZ were added to separate wells. After an additional 18 h incubation time the medium was collected and analysed. In some experiments cells were lysed and analysed for phosphoprotein activity.

#### Protein kinase assays

The binding affinities of MBZ were tested in a binding assay (DiscoverX, CA) against DYRK1B kinase tagged with DNA. Compounds that bind the kinase active site and directly or indirectly prevent kinase binding to an immobilised ligand, will reduce the amount of kinase captured on solid support. Conversely, test molecules that do not bind the kinase have no effect on the amount of kinase captured on the solid support. Binding constants were determined with this method using 11-point threefold serial dilutions of the compound. Curves were fitted using a non-linear least square t with the Levenberg–Marquardt algorithm [18]. Inhibition of DYRK1B activity was performed by Reaction labs, USA. Briefly, compounds were tested in 10-dose IC_50_ mode with threefold serial dilution starting at 10 μM and reactions were carried out at 10 μM ATP. Data are presented as % enzyme activity (relative to DMSO controls).

#### Measurement of M1 and M2 surface marker expression

Surface markers was measured as previously described [15]. THP-1 cells were incubated for 24 h with PMA, DMSO and AZ191. The cells were subsequently washed with cell culture media and incubated for an additional 24 h. Cells were then detached and mixed with the conjugated antibodies CD80-PC7 and CD163-PE (Beckman Coulter, Indianapolis, IN, USA). After 15 min, cells were washed with PBS (Sigma) and subsequently analysed on a Navios flow cytometer (Beckman Coulter) using the Kaluza software (Beckman Coulter).

#### Measurement of cytokines and phosphoproteins

Cytokines and phosphoproteins were measured using the Luminex/MAGPIX system and commercially available kits (Biorad, Hercules, CA) and were performed as described previously [15] and according to the instructions from the manufacturer. Briefly, for the cytokine assay supernatant samples were incubated with beads, detection antibody and streptavidin-PE. The fluorescence was subsequently measured using the MAGPIX instrument. For the phosphoprotein assay cell lysates were used and protein concentrations were determined using a Micro-BCA method (ThermoFischer Scientific, Waltham, MA, USA) to obtain equal amounts of samples in the assay.

#### Statistical analysis

Statistical analysis was performed using the Students t-test module in GraphPadPrism (GraphPad Software, San Diego, CA).

### Results and discussion

**Fig. 1:**
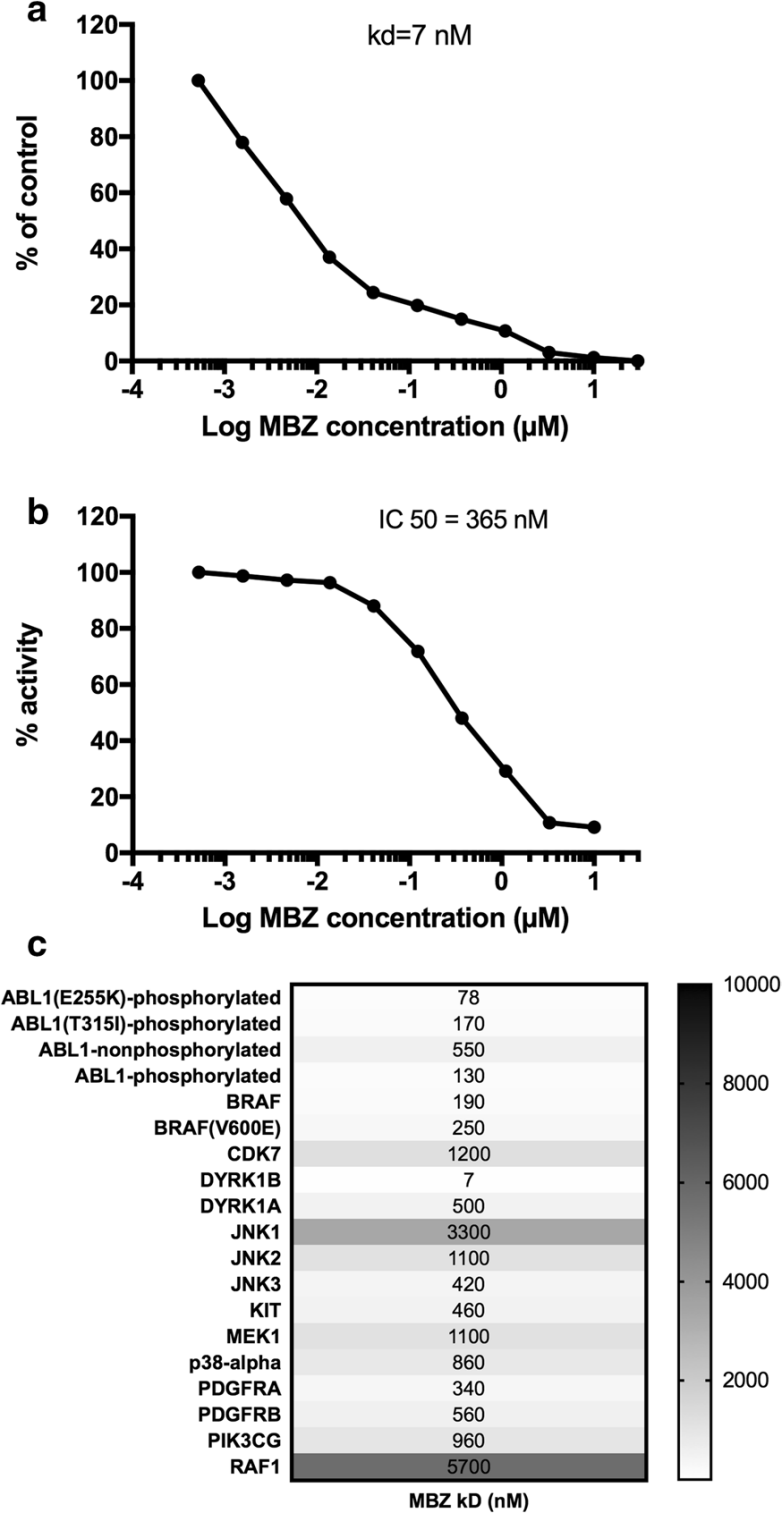
Inhibition of binding (**a**) and enzyme activity (**b**) of DYRK1B by MBZ. Measurements was performed by DiscoverX and Reactionlabs as detailed in methods. The Kd measurement was repeated with identical results. In **c** Kd determinations for other kinases analysed in parallel are shown

We previously showed that MBZ selectively and potently binds to several protein kinases [7]. When repeating and extending the initial kinase screen we noted that MBZ affected the dual specificity tyrosine phosphorylation-regulated kinase 1B (DYRK1B). In concentration–response experiment for binding affinity to DYRK1B, MBZ showed a kD of 7 nM and potently inhibited the kinase activity, with an IC_50_ of 360 nM (Fig. 1a, b). This should be compared with binding data (kD) for other hit kinases from the screening of the Scanedge and Kinomescan 456 from DiscoverX (Fig. 1c). The Kd of 7 nM for DYRK1B clearly stands out being considerably lower than for other kinases including ABL, BRAF and DYRK1A (Fig. 1c). The 7 nM Kd for MBZ binding to DYRK1B is a low concentration, compared with MBZ inhibition of tubulin polymerisation, which requires concentrations exceeding 1 µM [15]. These results are in accordance with validated results from the RepurposeVS, a drug repurposing computational platform [19]. DYRK1B is a serine/threonine kinase that is widely expressed in various cells especially in nondividing cells such as myoblasts that exit the cellcycle to terminally differentiate. DYRK1B controls the cellular change from a noncycling G0 state to S phase and also diminish oxidative stress by reducing intracellular levels of reactive oxygen species (ROS) [20]. Furthermore, DYRK1B is overexpressed in a subset of cancers and maintains cellular quiescence which increases cell survival in e.g. ovarian-, pancreatic-, and colon cancer [21–23].

**Fig. 2:**
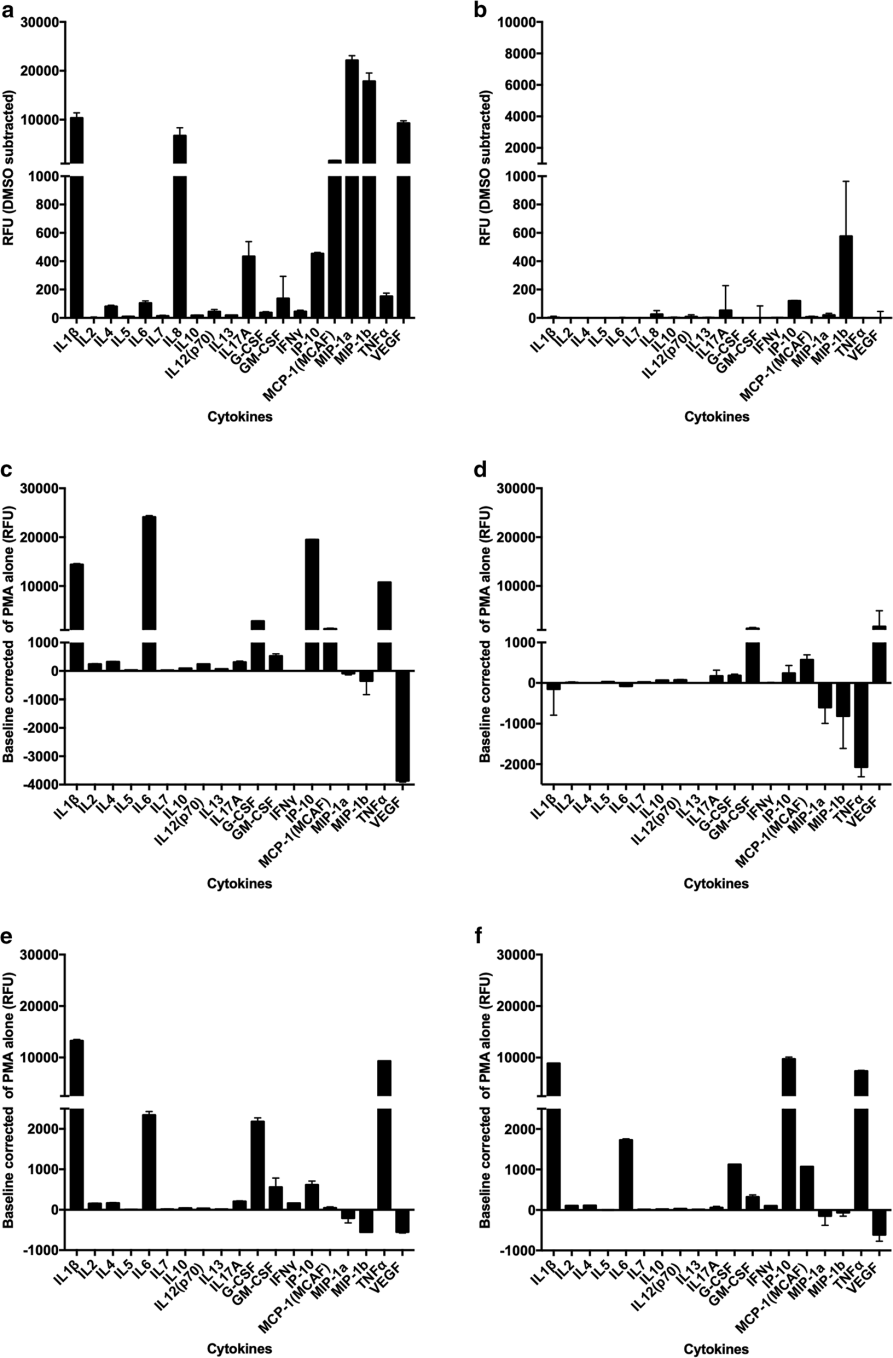
Cytokine release from monocytoid THP-1 cells in response to 24 h exposure to MBZ (**a**) and AZ191 (**b**). Corresponding cytokine release from PMA differentiated THP-1 macrophages in response to M1 stimuli LPS/IFNγ (**c**), M2 stimuli IL4/IL13 (**d**), MBZ 10 µM (**e**) and AZ191 10 µM (**f**) after 48 h of incubation (30 h PMA + 18 h with added stimuli and drugs). The results are expressed as relative fluorescence units (RFU) and presented as mean values ± standard deviation for duplicate measurements. Measurements was performed by a Luminex MAGPIX instrument and a multiplex kit from Biorad. The measurements of selected cytokines (IL1β and TNF) were repeated in 4 independent experiments with similar results. See “Results” for details

Next we tested the effect of MBZ and the selective inhibitor of DYRK1B, AZ191 [24], on cytokine release from THP-1 monocytes. AZ191 did not mimic the cytokine release profile induced by MBZ in untreated THP-1 monocytes (Fig. 2a, b) and only a small effect on IL1β secretion was noted when the cells were primed with LPS (Additional file 1: Fig. S1). In contrast, MBZ at 10 µM strongly induced cytokine release in these cells and in lower concentrations (1 µM) the effect on IL1β secretion could be clearly potentiated by priming with LPS (Fig. 2a and Additional file 1: Fig. S1).

**Fig. 3:**
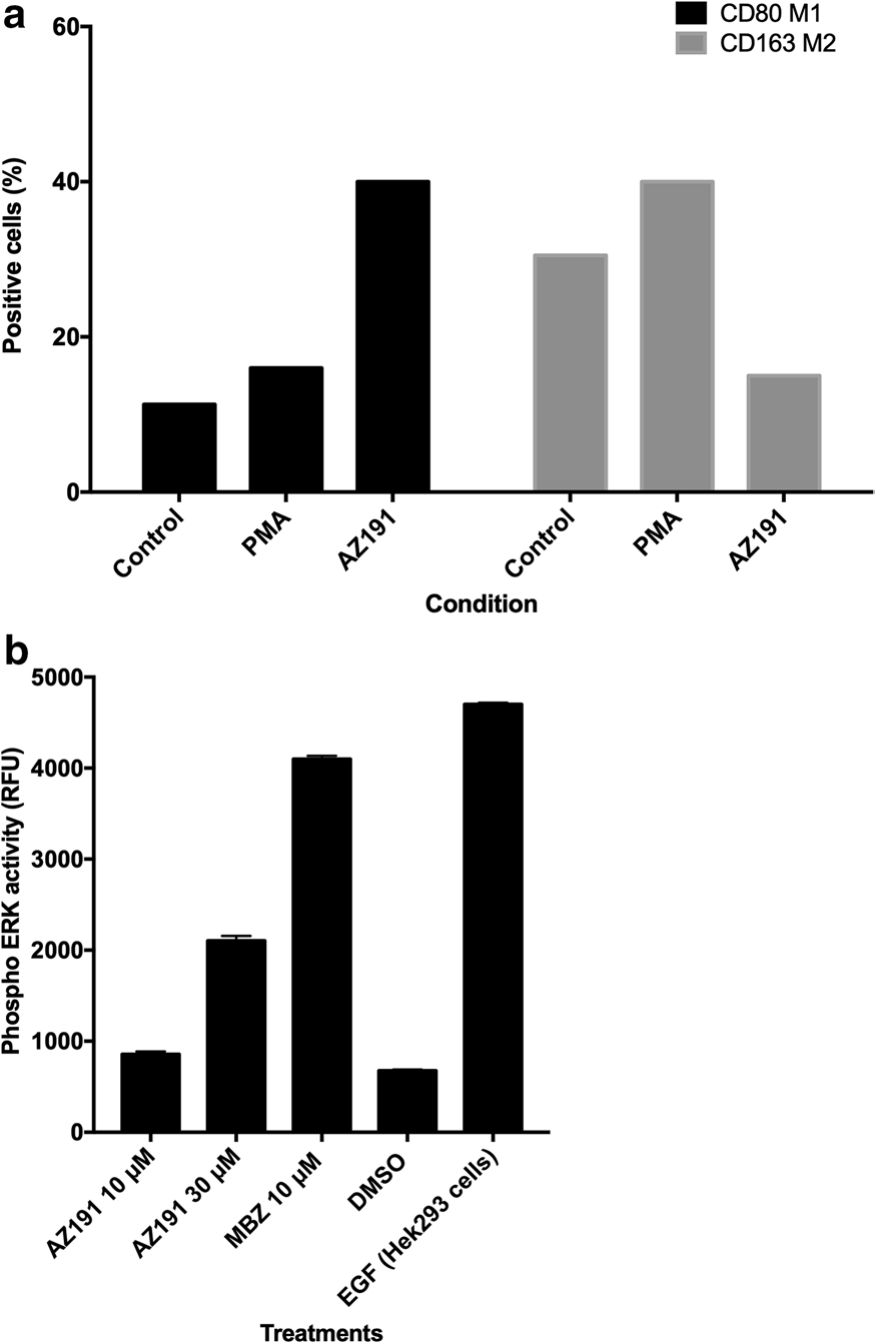
Flow cytometry analysis of CD80 and CD163 expression in response to 10 µM AZ191 (**a**) after 48 h of incubation. In **b** the effect of AZ191 and MBZ on ERK phosphoprotein activation in PMA differentiated THP-1 cells is shown. ERK activity was measured after 1 h exposure of the drugs. The results are expressed as relative fluorescence units (RFU) and presented as mean values ± standard deviation for duplicate measurements. See “Methods” for details. Measurements were performed by a Luminex Magpix instrument and a ERK phosphoprotein kit from Biorad. A lysate of EGF-treated HEK293 cells provided in the kit served as positive control

In contrast, in THP-1 cells differentiated to macrophages by PMA, AZ191 induced a M1-like pro-inflammatory cytokine release pattern similar to MBZ and LPS/IFNγ (Fig. 2c–f). MBZ (Fig. 2e), LPS/IFNγ (Fig. 2c) and AZ191 (Fig. 2f) clearly increased both IL1β and TNF (tumour necrosis factor) and decreased VEGF (vascular endothelial growth factor) opposite to the effect of M2 inducing IL13/IL4 stimulation (Fig. 2d). In repeat experiments with IL1β and TNF the effect of AZ191 was statistically significant compared to DMSO control (P < 0.01, n = 4, paired Students t-test). Moreover, AZ191 increased the expression of the M1 marker CD80 and decreased the M2 marker CD163 in the THP-1 macrophage model (Fig. 3a with the original flow cytometry histograms shown in Additional file 2: Fig. S2), corroborating previously published results for MBZ [15].

The difference between THP-macrophages and THP-1 monocytes in response to AZ191 and MBZ is presently unexplained. Monocytoid THP-1 cells are often used as surrogate model for dendritic cells (DCs) in evaluation of allergen potential of small molecules [25–27]. MBZ-induced cytokine release in naive THP-1 cells is dominated by IL1β and IL8, a response considered to reflect maturation of immature DCs to active functional antigen-presenting DCs [25, 28]. Based on the expression profile for 13 gene markers for DC activation, the monocytic THP-1 cells significantly more closely resembled the human DC model compared to THP-1 differentiated macrophages [25]. On the other hand, THP-1 macrophages have been reported to closely mimic the phenotype of human macrophages [29]. Thus, these cellular models reflecting DCs and macrophages are different and this may also apply to signaling pathways.

One consequence of DYRK1B inhibition is ERK (extracellular signal-regulated kinase) activation and it has previously been shown that MBZ strongly induces ERK activation [30]. Indeed, although less pronounced than for MBZ, AZ191 dose-dependently increased phospho-ERK activity in THP-1 macrophages (Fig. 3b). Previous studies have demonstrated that IL1β release require two signals. The first is provided by toll-like-receptor-mediated NF-kappaB activation whereas the second can be mediated by danger signals such as stimulation of ATP receptors or other stimuli including elevated ROS production or perturbation of lysosome integrity [15]. In this study it was demonstrated that high dose MBZ (> 3 µM) activated both signals, the first via TLR8 stimulation, whereas low concentrations (1 µM) only could activate the second one [15]. From the data presented here one can speculate that ERK activation induced by MBZ and AZ191 might be the second signal in PMA differentiated THP-1 macrophages. However, it should be noted that the present results are only based on one cell line model only and the relevance to the human immune system remains to be established.

Monocytes and macrophages may express at least two different phenotypes which can be either pro-inflammatory (M1) or anti-inflammatory (M2) [17, 31, 32]. The M1 monocytes/macrophages is characterised by tumor-suppressive properties such as production of cytokines that activate T-cells and can also induce direct cytotoxic anti-tumour effects. The M2 counterpart is the typical phenotype present in solid tumours and exert tumour-supporting functions such as immunosuppression and stimulation of angiogenesis [17, 31, 32]. Small molecules that have the capability to shift the polarisation from M2 to M1 would thus be potentially interesting for cancer therapy. This type of drugs could also be fruitfully combined with other immunotherapy, such as checkpoint inhibitors or CAR T cells. If MBZ is such a drug will require further investigation.

## Limitations

The work is restricted only to cell line models.

## Additional files

**Additional file 1: Fig. S1.** The effect of AZ191 and MBZ with and without LPS on IL-1 release in THP-1 monocytes is shown.

**Additional file 2: Fig. S2.** Original flow cytometry histograms from Fig. 3 is shown.

## Abbreviations

MBZ: mebendazole
Kd: dissociation constant
IC50: inhibitory concentration 50%
DYRK1B: dual specificity tyrosine-phosphorylation-regulated kinase 1B
LPS: lipopolysaccharide
IFNγ: interferon gamma
PBMC: peripheral blood mononuclear cells
IL13: interleukin-13
PMA: phorbol-12-myristate-13-acetate
IL4: interleukin-4
DMSO: dimethylsulfoxide
ABL: Bcr-Abl tyrosine-kinase
BRAF: serine/threonine-protein kinase B-Raf
ROS: reactive oxygen species
IL1β: interleukin-1 beta
TNF: tumor necrosis factor
VEGF: vascular endothelial growth factor
DC: dendritic cell
IL18: interleukin-18
ERK: extracellular signal-regulated kinase
CAR T: chimeric antigen receptor T cells

## References

[1] Pantziarka P, Bouche G, Meheus L, Sukhatme V, Sukhatme VP (2014). Repurposing Drugs in Oncology (ReDO)—mebendazole as an anti-cancer agent. Ecancermedicalscience.

[2] Doudican N, Rodriguez A, Osman I, Orlow SJ (2008). Mebendazole induces apoptosis via Bcl-2 inactivation in chemoresistant melanoma cells. Mol Cancer Res.

[3] Bai R-Y, Staedtke V, Aprhys CM, Gallia GL, Riggins GJ (2011). Antiparasitic mebendazole shows survival benefit in 2 preclinical models of glioblastoma multiforme. Neuro-oncology..

[4] Sasaki J-I, Ramesh R, Chada S, Gomyo Y, Roth JA, Mukhopadhyay T (2002). The anthelmintic drug mebendazole induces mitotic arrest and apoptosis by depolymerizing tubulin in non-small cell lung cancer cells. Mol Cancer Ther.

[5] Martarelli D, Pompei P, Baldi C, Mazzoni G (2008). Mebendazole inhibits growth of human adrenocortical carcinoma cell lines implanted in nude mice. Cancer Chemother Pharmacol.

[6] Mukhopadhyay T, Sasaki J-I, Ramesh R, Roth JA (2002). Mebendazole elicits a potent antitumor effect on human cancer cell lines both in vitro and in vivo. Clin Cancer Res.

[7] Nygren P, Fryknäs M, Agerup B, Larsson R (2013). Repositioning of the anthelmintic drug mebendazole for the treatment for colon cancer. J Cancer Res Clin Oncol.

[8] Doudican NA, Byron SA, Pollock PM, Orlow SJ (2013). XIAP downregulation accompanies mebendazole growth inhibition in melanoma xenografts. Anticancer Drugs.

[9] Bai R-Y, Staedtke V, Rudin CM, Bunz F, Riggins GJ (2015). Effective treatment of diverse medulloblastoma models with mebendazole and its impact on tumor angiogenesis. Neuro Oncol.

[10] Bodhinayake I, Symons M, Boockvar JA (2015). Repurposing mebendazole for the treatment of medulloblastoma. Neurosurgery..

[11] Pinto LC, Soares BM, Pinheiro JD, Riggins GJ, Assumpção PP, Burbano RM, Montenegro RC (2015). The anthelmintic drug mebendazole inhibits growth, migration and invasion in gastric cancer cell model. Toxicol In Vitro.

[12] Dobrosotskaya IY, Hammer GD, Schteingart DE, Maturen KE, Worden FP (2011). Mebendazole monotherapy and long-term disease control in metastatic adrenocortical carcinoma. Endocr Pract..

[13] Larsson R, Nygren P (2013). Drug repositioning from bench to bedside: tumour remission by the antihelmintic drug mebendazole in refractory metastatic colon cancer. Acta Oncol.

[14] Larsen AR, Bai R-Y, Chung JH, Borodovsky A, Rudin CM, Riggins GJ (2015). Repurposing the antihelmintic mebendazole as a hedgehog inhibitor. Mol Cancer Ther..

[15] Blom K, Senkowski W, Jarvius M, Berglund M, Rubin J, Lenhammar L (2017). The anticancer effect of mebendazole may be due to M1 monocyte/macrophage activation via ERK1/2 and TLR8-dependent inflammasome activation. Immunopharmacol Immunotoxicol.

[16] Rubin J, Mansoori S, Blom K, Berglund M, Lenhammar L, Andersson C (2018). Mebendazole stimulates CD14+ myeloid cells to enhance T-cell activation and tumour cell killing. Oncotarget.

[17] Stewart DA, Yang Y, Makowski L, Troester MA (2012). Basal-like breast cancer cells induce phenotypic and genomic changes in macrophages. Mol Cancer Res.

[18] Davis MI, Hunt JP, Herrgard S, Ciceri P, Wodicka LM, Pallares G (2011). Comprehensive analysis of kinase inhibitor selectivity. Nat Biotechnol.

[19] Issa NT, Peters OJ, Byers SW, Dakshanamurthy S (2015). RepurposeVS: a drug repurposing-focused computational method for accurate drug-target signature predictions. Comb Chem High Throughput Screen.

[20] Becker W (2018). A wake-up call to quiescent cancer cells—potential use of DYRK1B inhibitors in cancer therapy. FEBS J.

[21] Hu J, Deng H, Friedman EA (2013). Ovarian cancer cells, not normal cells, are damaged by Mirk/Dyrk1B kinase inhibition. Int J Cancer.

[22] Ewton DZ, Hu J, Vilenchik M, Deng X, Luk K-C, Polonskaia A (2011). Inactivation of mirk/dyrk1b kinase targets quiescent pancreatic cancer cells. Mol Cancer Ther.

[23] Jin K, Ewton DZ, Park S, Hu J, Friedman E (2009). Mirk regulates the exit of colon cancer cells from quiescence. J Biol Chem.

[24] Ashford AL, Oxley D, Kettle J, Hudson K, Guichard S, Cook SJ (2013). A novel DYRK1B inhibitor, AZ191, demonstrates that DYRK1B acts independently of GSK3β to phosphorylate cyclin D1 at threonine-286, not threonine-288. Biochem J.

[25] Lambrechts N, Verstraelen S, Lodewyckx H, Witters H, Nelissen I, Van Den Heuvel R (2009). THP-1 monocytes but not macrophages as a potential alternative for CD34+ dendritic cells to identify chemical skin sensitizers. Toxicol Appl Pharmacol.

[26] Corti D, Galbiati V, Gatti N, Marinovich M, Galli CL, Corsini E (2015). Optimization of the THP-1 activation assay to detect pharmaceuticals with potential to cause immune mediated drug reactions. Toxicol In Vitro.

[27] Galbiati V, Papale A, Kummer E, Corsini E (2016). In vitro models to evaluate drug-induced hypersensitivity: potential test based on activation of dendritic cells. Front Pharmacol..

[28] Miyazawa M, Ito Y, Yoshida Y, Sakaguchi H, Suzuki H (2007). Phenotypic alterations and cytokine production in THP-1 cells in response to allergens. Toxicol In Vitro.

[29] Chanput W, Mes JJ, Wichers HJ (2014). THP-1 cell line: an in vitro cell model for immune modulation approach. Int Immunopharmacol.

[30] Gao J, Zhao Y, Lv Y, Chen Y, Wei B, Tian J (2013). Mirk/Dyrk1B mediates G0/G1 to S phase cell cycle progression and cell survival involving MAPK/ERK signaling in human cancer cells. Cancer Cell Int.

[31] Panni RZ, Linehan DC, DeNardo DG (2013). Targeting tumor-infiltrating macrophages to combat cancer. Immunotherapy..

[32] Fridlender ZG (2013). Modifying tumor-associated macrophages: an important adjunct to immunotherapy. Oncoimmunology..

