# Supplementary figures and images for: Mebendazole-induced M1 polarisation of THP-1 macrophages may involve DYRK1B inhibition
Source: BMC Res Notes. 2019 Apr 22;12:234. doi: 10.1186/s13104-019-4273-5 (PMC6477744; doi:10.1186/s13104-019-4273-5)

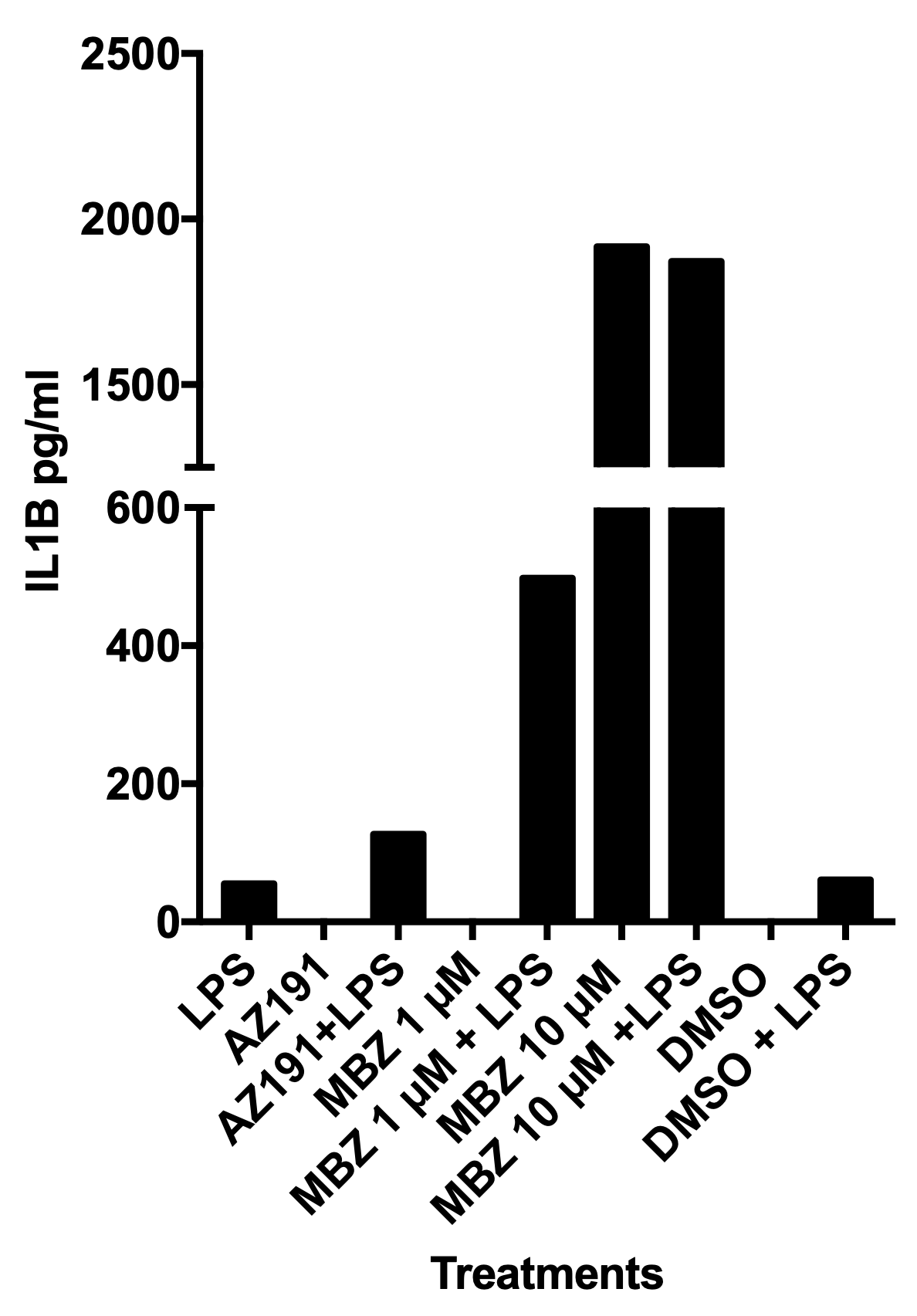

Supplement: Supplementary file 1 — Additional file 1: Fig. S1. The effect of AZ191 and MBZ with and without LPS on IL-1 release in THP-1 monocytes is shown. [file 13104_2019_4273_MOESM1_ESM.tiff]

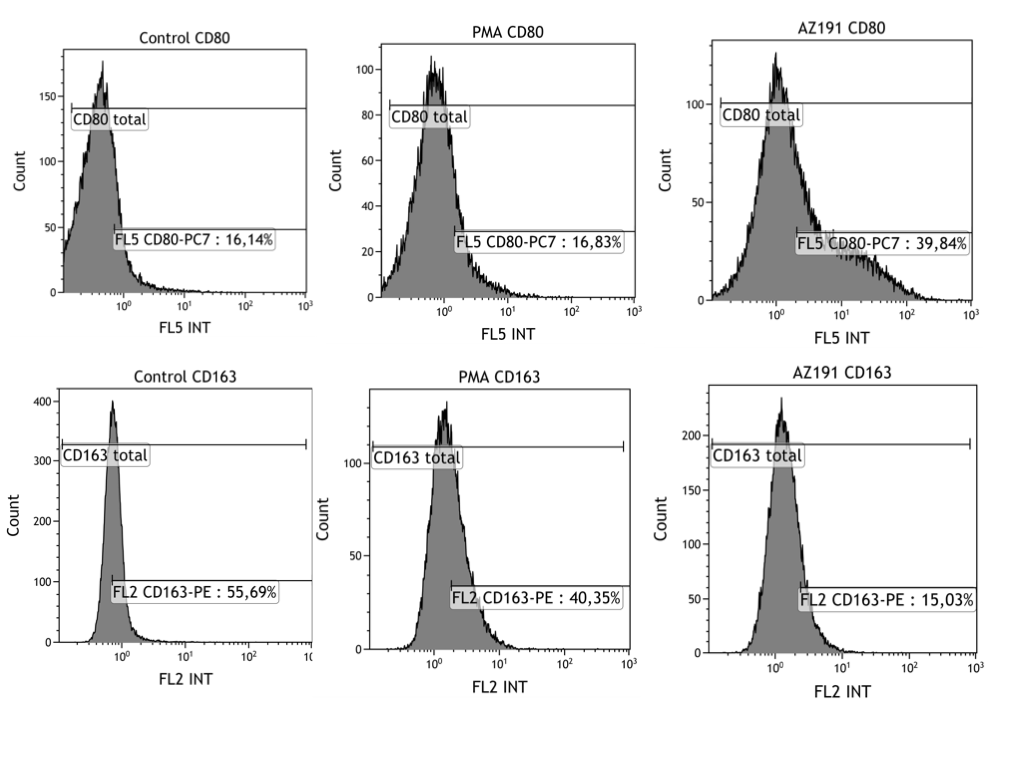

Supplement: Supplementary file 2 — Additional file 2: Fig. S2. Original flow cytometry histograms from Fig. 3 is shown. [file 13104_2019_4273_MOESM2_ESM.tiff]
